# Melanoma stimulates the proteolytic activity of HaCaT keratinocytes

**DOI:** 10.1186/s12964-022-00961-w

**Published:** 2022-09-19

**Authors:** Justyna Mazurkiewicz, Aleksandra Simiczyjew, Ewelina Dratkiewicz, Magdalena Kot, Katarzyna Pietraszek-Gremplewicz, Dominika Wilk, Marcin Ziętek, Rafał Matkowski, Dorota Nowak

**Affiliations:** 1 Department of Cell Pathology, Faculty of Biotechnology, University of Wroclaw, Joliot-Curie 14a, 50-383 Wrocław, Poland; 2 Department of Oncology and Division of Surgical Oncology, Wroclaw Medical University, Plac Hirszfelda 12, 53-413 Wrocław, Poland; 3 Wroclaw Comprehensive Cancer Center, Plac Hirszfelda 12, 53-413 Wrocław, Poland

**Keywords:** HaCaT, Melanoma, Tumor microenvironment, Proteolysis, Migration, Invasion

## Abstract

**Background:**

Keratinocytes constitute a major part of the melanoma microenvironment, considering their protective role towards melanocytes in physiological conditions. However, their interactions with tumor cells following melanomagenesis are still unclear.

**Methods:**

We used two in vitro models (melanoma-conditioned media and indirect co-culture of keratinocytes with melanoma cells on Transwell inserts) to activate immortalized keratinocytes towards cancer-associated ones. Western Blotting and qPCR were used to evaluate keratinocyte markers and mediators of cell invasiveness on protein and mRNA expression level respectively. The levels and activity of proteases and cytokines were analysed using gelatin-FITC staining, gelatin zymography, chemiluminescent enzymatic test, as well as protein arrays. Finally, to further study the functional changes influenced by melanoma we assessed the rate of proliferation of keratinocytes and their invasive abilities by employing wound healing assay and the Transwell filter invasion method.

**Results:**

HaCaT keratinocytes activated through incubation with melanoma-conditioned medium or indirect co-culture exhibit properties of less differentiated cells (downregulation of cytokeratin 10), which also prefer to form connections with cancer cells rather than adjacent keratinocytes (decreased level of E-cadherin). While they express only a small number of cytokines, the variety of secreted proteases is quite prominent especially considering that several of them were never reported as a part of secretome of activated keratinocytes’ (e.g., matrix metalloproteinase 3 (MMP3), ADAM metallopeptidase with thrombospondin type 1 motif 1). Activated keratinocytes also seem to exhibit a high level of proteolytic activity mediated by MMP9 and MMP14, reduced expression of TIMPs (tissue inhibitor of metalloproteinases), upregulation of ERK activity and increased levels of MMP expression regulators-RUNX2 and galectin 3. Moreover, cancer-associated keratinocytes show slightly elevated migratory and invasive abilities, however only following co-culture with melanoma cells on Transwell inserts.

**Conclusions:**

Our study offers a more in-depth view of keratinocytes residing in the melanoma niche, drawing attention to their unique secretome and mediators of invasive abilities, factors which could be used by cancer cells to support their invasion of surrounding tissues.

**Video abstract**

**Supplementary Information:**

The online version contains supplementary material available at 10.1186/s12964-022-00961-w.

## Background

The topic of the impact of the tumor microenvironment (TME) on the progression of various types of cancer has attracted more and more attention in recent years. The melanoma niche is composed of cellular components such as keratinocytes, cancer-associated fibroblasts, adipocytes, and immune cells as well as factors such as state of hypoxia or extracellular acidification [1–3]. Each element of TME seems to play a different role in cancer progression. Cancer-associated fibroblasts constitute a major element of the melanoma microenvironment [4]. They fulfill diverse functions such as production of extracellular matrix components and secretion of proinflammatory and angiogenic factors [5, 6]. Our previous research showed that in comparison to normal fibroblasts melanoma-associated fibroblasts exhibit increased migration, invasion, and proteolytic activity, which in turn might affect melanoma invasion [7]. Adipocytes on the other hand, are considered as a source of high energetic compounds and metabolites, which may be used by cancer cells for rapid proliferation [8, 9]. Among the cellular components of the tumor niche, we can also distinguish immune cells such as natural killer cells, T lymphocytes, B lymphocytes, dendritic cells, macrophages and myeloid-derived suppressor cells [10, 11]. At tumor onset, immune cells exhibit anti-cancer properties. However, in advanced stages of cancer, cells which support cancer progression, e.g., tumor-associated macrophages are observed [11, 12]. The role of another essential component of the melanoma microenvironment – keratinocytes, which are one of the main cell types in the epidermis [13], seems to be the least studied.

In physiological conditions, keratinocytes control proliferation of melanocytes through direct interactions or in a paracrine manner. As many as thirty-six keratinocytes can be connected to one melanocyte [3, 14]. Direct interactions between these two types of cells are mainly based on E-cadherin-dependent cell to cell adhesion [15]. During melanoma development, the level of E-cadherin decreases, while the level of N-cadherin increases, leading to the release of melanocytes from the control of keratinocytes. Consequently, melanocytes start to form connections with cells that express N-cadherin, such as fibroblasts [16]. The switch of cadherin level is triggered by epithelial-mesenchymal transition regulators (e.g., Snail, Slug, Twist) and may stimulate melanoma tumorigenesis [15]. Furthermore, Hsu et al. have shown that restoration of E-cadherin expression in melanoma cells leads to their reconnection to keratinocytes, as well as decreased melanoma cell growth and colony formation [17]. Moreover, under UV radiation keratinocytes induce DNA damage repair pathways in pigmented cells via secretion of α-melanocortin and endothelin-1 (End-1), thus protecting melanocytes from transforming into melanoma [18].

On the other hand, some evidence points to the possible pro-tumorigenic role of keratinocytes in the melanoma niche. Jamal et al. have shown that endothelin-1, which is secreted by keratinocytes, activates caspase 8. This protein, in turn, transiently associates to E-cadherin/catenin complex in melanoma cells resulting in E-cadherin downregulation and, thus, stimulates cancer cell invasion. The mechanism of End-1 dependent caspase 8 activation remains unclear [19]. In addition, Smith et al. indicated that extracellular End-1 promotes extracellular-signal-regulated kinase (ERK) phosphorylation following protein kinase C (PKC) activation in serine/threonine-protein kinase B-Raf (BRAF) inhibitor treated cells. Moreover, expression of End-1 depends on melanocyte inducing transcription factor (MITF) level, depletion or overexpression of which led to either End-1 downregulation or enhanced production, respectively. Inhibition of endothelin receptor B led to enhanced sensitivity of melanoma cells resistant to BRAF inhibitors [20, 21]. Furthermore, keratinocytes respond to fibroblast-derived keratinocyte growth factor (KGF) with the secretion of c-KIT ligand—stem cell factor (SCF). SCF in turn activates the Akt and mitogen-activated protein kinase (MAPK) pathways responsible for controlling tumor cell invasion and proliferation [22].

There are only a few reports about the effects of melanoma on keratinocytes. One of them is concerning the switch in the level of selected cytokeratins, cytoplasmic intermediate filament proteins, which are differentiation status indicators of keratinocytes [23, 24]. Cytokeratin 10 (CK10) is highly expressed in suprabasal keratinocytes that undergo cornification and shedding. Cytokeratin 14 in turn, is characteristic of rapidly proliferating basal keratinocytes. As described by Kodet et al., keratinocytes under the influence of melanoma have changed cytokeratin expression profiles. They exhibited downregulation of CK10 and upregulation of CK14 [24].

Cellular elements of the tumor microenvironment are known to be recruited by cancer cells. The impact of melanoma on a few cell types present in the tumor microenvironment is intensively studied [25, 26], however the influence of melanoma on keratinocytes remains unclear. Taking into consideration research indicating the inhibitory effect of keratinocytes on melanocytes and their supporting role in melanoma invasion and the acquisition of resistance we decided to evaluate the changes in keratinocytes that might be triggered by melanoma.

## Methods

### Cell culture

Cancer-associated keratinocytes were obtained from immortalized keratinocytes – HaCaT (Cell Lines Service) using four different melanoma cell lines: A375 and Hs294T purchased from American Type Culture Collection and WM1341D and WM9 cell lines obtained from Rockland Immunochemicals, Inc. These cell lines differ in their origin – WM1341D and A375 are derived from the primary tumor, while WM9 and Hs294T are metastatic ones. They also possess distinct invasive abilities, which WM1341D characterized by low invasion rate, whereas A375, WM9, and Hs294T being highly invasive cell lines.

HaCaT keratinocytes were grown in DMEM (Dulbecco’s Modified Eagle Medium, Gibco; 4.5 g/l glucose, 3.7 g/l NaHCO_3_, L-glutamine) supplemented with 10% fetal bovine serum (FBS, Gibco) and antibiotics (10,000 U/ml penicillin, 10 mg/ml streptomycin, 25 µg/ml amphotericin B, Gibco). Cells were passaged twice a week before they reached 80% confluency. After the medium was aspirated, cells were washed and then incubated with 0.05% EDTA (Invitrogen) for 10 min at 37 °C followed by the incubation with a freshly prepared mixture of 0.05% trypsin (without EDTA) (Gibco) and 0.025% EDTA (Invitrogen) for 3 min at 37 °C. Cells were then collected in a complete culture medium and centrifuged for 5 min at 300×*g*.

Melanoma cells were cultured in DMEM (IITD PAN, Wrocław, Poland) containing 4.5 g/l glucose and 1.5 g/l NaHCO_3_ supplemented with 10% fetal bovine serum (FBS), 2 mM glutamine (Gibco) and antibiotics (10,000 U/ml penicillin, 10 mg/ml streptomycin, 25 µg/ml amphotericin B). Cells were cultured in 25 cm^2^ tissue culture flasks (Nest) at 37 °C in 5%CO_2_/95% humidified air and passaged twice a week using 0.25% trypsin/0.05% EDTA solution (IITD PAN, Wrocław, Poland).

### Acquisition of the melanoma-conditioned medium

Melanoma cells were seeded into 75 cm^2^ tissue culture flasks. Upon reaching 70–80% confluence, cells were washed three times with phosphate-buffered saline (PBS) and medium without FBS was added. After 72 h medium was collected, centrifuged for 15 min at 1000×*g* and frozen at −20 °C. Media harvested from at least three biological replicates were thawed, mixed, filtered through 0.22 µm filters (Nest), aliquoted and stored at −20 °C.

### CAKs acquisition

**Fig. 1:**
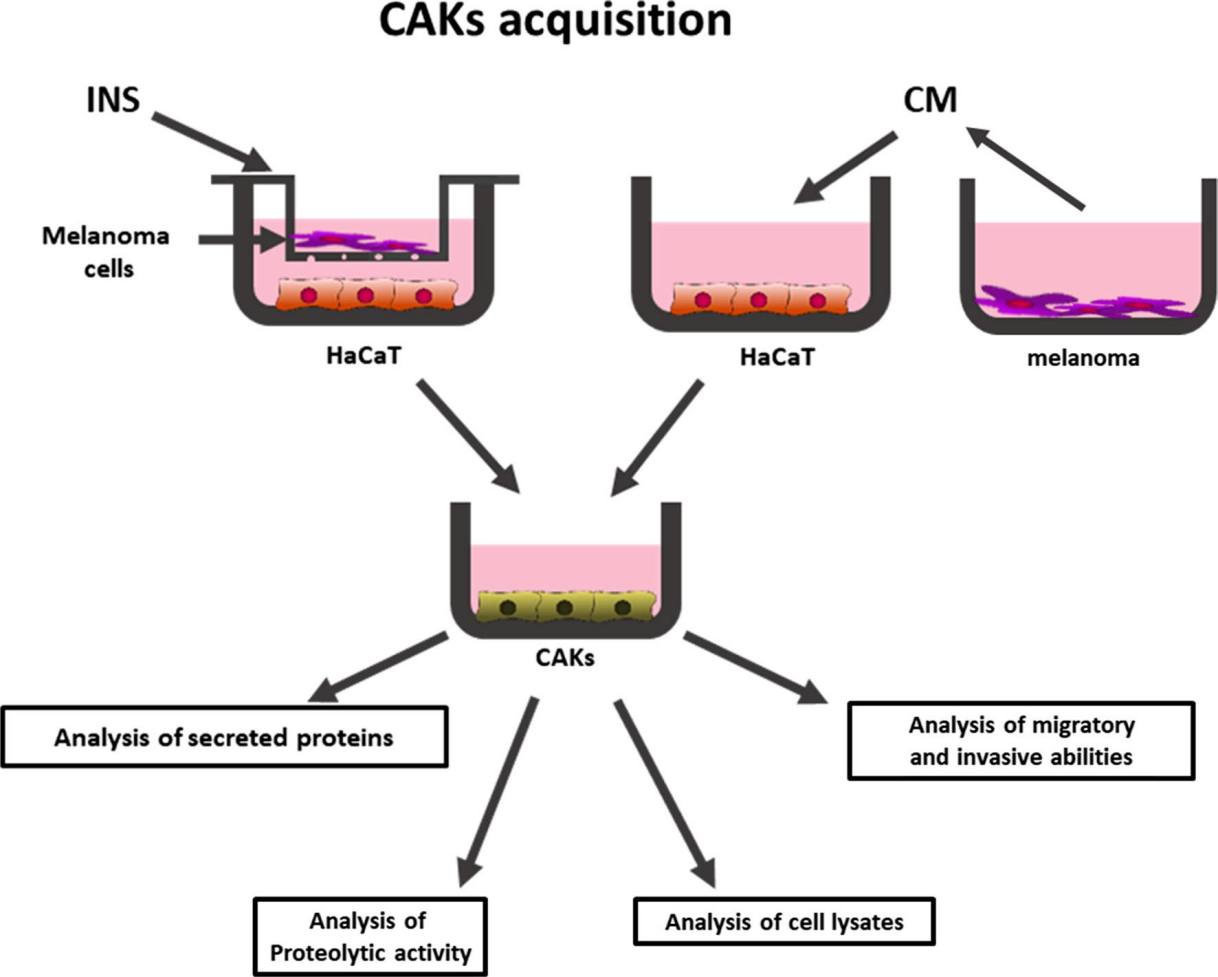
Methods of cancer associated keratinocytes (CAKs) acquisition. HaCaT cells were cultured in the presence of melanoma on Transwell inserts (INS) or with melanoma conditioned media (CM). After seven days of incubation cells were used for further experiments

Cancer-associated keratinocytes (CAKs) were acquired from HaCaT by culturing them in the presence of melanoma-conditioned media or in indirect co-culture with melanoma cells using Transwell inserts (0.4 μm pores, Falcon) (Fig. 1). Keratinocytes were grown in these conditions for seven days, then they were collected and used for further experiments or – for secreted protein expression analysis – cells were washed three times with PBS and the culture media was changed to the fresh ones without FBS for another 72 h. Next, the media was aspirated, centrifuged for 15 min at 1000×*g* and frozen at −20 °C.

Considering that in both systems, media had to be suitable for two different types of cells (keratinocytes and melanoma cell lines), we used a combination of DMEM (suitable for keratinocytes, kDMEM) and DMEM (appropriate for melanoma cells, mDMEM) in 1:1 ratio, therefore control for each experiment constituted keratinocytes cultured in such a medium.

### Western blot analysis

CAKs were obtained as previously described in the CAKs acquisition section. Following, cells were transferred on ice, washed three times with PBS and lysates were collected in urea buffer (50 mM Tris, pH 7.4, 5% SDS, 8.6% sucrose, 74 mM urea, 1 mM dichlorodiphenyltrichloroethane), supplemented with protease and phosphatase inhibitors cocktails (Sigma). Media used for secreted protein level analysis were acquired as described in the CAKs acquisition section and concentrated using Amicon® Ultra-4 Centrifugal Filters (Merck Millipore). Protein concentration was estimated using the standard BCA (Bicinchoninic acid) procedure (Thermofisher). Samples with normalized level of protein (5 μg of conditioned media and 10 μg of cell lysates) were separated by 10% polyacrylamide gel electrophoresis in the presence of sodium dodecylsulfate (SDS-PAGE) as described by Laemmli [27] and transferred onto nitrocellulose membranes, as reported by Towbin et al. [28]. Primary antibodies against CK14 (Abcam), CK10 (Abcam), E-cadherin (Cell Signaling), MMP9 (Abcam), pERK (Cell Signalling), ERK (Cell Signalling), pAkt (Cell Signalling), Akt (Cell Signalling), RUNX2 (Santa Cruz), Gal-3 (Santa Cruz) as well as secondary goat anti-mouse and anti-rabbit antibodies conjugated with horseradish peroxidase (Cell Signaling) were used according to the manufacturer’s protocols. Blots were developed with the Clarity Western ECL Substrate (Bio-Rad), then scanned using ChemiDoc machine (Bio-Rad). Analysis was conducted using ImageLab Software (ver. 6.0, Bio-Rad). At least three independent experiments were performed. Results were normalized to Ponceau S staining.

### 2D wound healing

Cells were placed into ImageLock 96-well plates (IncuCyte ImgeLock, Sartorius), which were previously coated with Matrigel (1 mg/ml). After 24 h, in all wells the standardized scratches were made using the Wound Maker™ (Essen Bioscience). Cells were then washed with PBS and fresh medium was added into each well. Phase-contrast time-lapse images were made with a time interval of 2 h, using a 10× objective in an IncuCyte® Live-Cell Analysis System. Cells were allowed to migrate and cover the wound for 36 h. Representative results were analysed using IncuCyte® Scratch Wound Cell Migration Software Module (Sartorius). The relative wound density represents the increase in the area covered by the cells over time. Three biological repetitions of the experiments, each condition consisting of three replicates, were conducted.

### Transwell invasion assay

To evaluate cell invasive potential, we used Transwell filters (8 µm pores, Falcon) placed in 24-well plates. CAKs acquired as previously mentioned were starved for 24 h in a high glucose DMEM medium without FBS. Further, cells were seeded in a medium without FBS onto each Transwell filter, which was coated with Matrigel (1 mg/ml). Medium containing 20% FBS constituted a chemoattractant at the bottom of each well. After 24 h incubation, the layer of Matrigel on the top of the filter with the cells that didn’t invade was removed. Cells that invaded through the membranes were fixed with 4% formaldehyde, their nuclei were visualized with Hoechst 33342 staining and cells were counted using fluorescent microscopy. The results of experiments are shown as a relative invasion factor (%). The number of control cells which invaded through the Transwell filters constitutes 100%. The experiments were conducted three times, each condition was executed in triplicate.

### Proliferation assay

To evaluate the cell proliferation rate, The Cell Proliferation Kit II (XTT) (Roche) was utilized according to the manufacturer’s protocol. Briefly, CAKs were placed in 96-well plates, and the XTT labeling mixture was added into each well at time 0 (T0) and after 24 h (T24) of cell growth. The absorbance at 450 nm was measured after 3 h of incubation at 37 °C. The results were background corrected. The mean proliferation rate was calculated based on the absorbance values acquired for 24 h relative to T0. The proliferation of control cells was set as 100%. The experiments were performed on at least three biological repetitions in triplicate for each condition.

### Fluorescent-gelatin degradation assay

Fluorescent-gelatin degradation assays were performed according to the protocol described by Mazurkiewicz et al. [29]. Poly-L-lysine-coated coverslips (Corning) were washed with PBS and fixed with 0.5% glutaraldehyde for 15 min. Further, the coverslips were placed on a 30 μl drop of fluorescein (FITC)-labeled gelatin, incubated for 10 min, and rinsed with PBS. Then, the residual reactive groups were quenched with sodium borohydride (5 mg/ml). Cells were trypsinized, seeded in 24-well plates on glass coverslips, and incubated at 37 °C for 16 h. Next, cells were fixed with 4% formaldehyde, permeabilized with 0.1% Triton X-100, and stained with Alexa Fluor 568 phalloidin to visualize filamentous actin. Leica SP8 confocal microscope with LAS X software (ver. 3.3.0, Leica) was used for image capture. Places of degraded gelatin were visible as dark holes (the absence of fluorescence) in the green, fluorescent gelatin matrix.

### MMP14 activity assay

To assess MMP14 activity the SensoLyte 520 MMP14 Assay Kit (AnaSpec) was used. Cells were seeded into 6-well plates and activated towards CAKs as previously described. After seven days of keratinocytes incubation with melanoma CM or with melanoma on Transwell inserts, cells were rinsed with PBS, harvested in assay buffer containing 0.1% Triton-X 100 and incubated for 10 min at 4 °C. Samples were then centrifuged for 10 min at 4 °C at 2500×*g*, and supernatants were transferred into fresh tubes. Protein concentration was evaluated using a standard BCA assay. Samples with the same amount of protein (30 µg) were incubated with an activator for 2.5 h at 37 °C. Next, the substrate was added and the enzymatic reaction was conducted for 30 min at 37 °C and stopped with a stop solution. The fluorescence of the product was measured (Ex/Em = 475/500 nm) using a GloMax Discover plate reader (Promega). Control was set as 100% of MMP14 activity. Experiments were performed three times, each condition in two replicates.

### Gelatin zymography

The proteolytic activity of gelatinases in CAKs culture media was determined using the gelatin zymography method. Keratinocytes were activated towards CAKs as previously described. Next, cells were washed with PBS and a fresh medium without FBS was added. After 72 h of incubation at 37 °C, the medium was collected and then concentrated using Amicon® Ultra-4 Centrifugal Filters (Merck Millipore). Next, protein concentration was evaluated using the standard BCA procedure and media were analysed on SDS–polyacrylamide gels containing gelatin (1 mg/ml). The gels were stained with Coomassie Brilliant Blue R-250 (Sigma). Places where gelatin was degraded by MMPs were identified as brighter stripes on gels. Images were captured using ChemiDoc (Bio-Rad) and densitometry of at least three biological repetitions was performed in ImageJ software.

### Human cytokine array

To detect the elements of CAKs secretome a Cytokine Array Kit (R&D systems) was utilized. It allows to identify 36 different cytokines and chemokines by using antibodies, which are immobilized on a nitrocellulose membrane. The experiment was conducted according to the protocol provided by the manufacturer, using conditioned media collected from: control cells and keratinocytes treated with CM from Hs294T. Samples with an identical amount of protein (10 µg) were mixed with a biotinylated detection antibodies cocktail and added onto nitrocellulose membranes containing primary antibody dots and incubated overnight. Next, the membranes were washed and the chemiluminescent signal was detected using streptavidin-HRP. A chemiluminescent signal was measured using ChemiDoc Imaging System (BioRad) and analyzed using ImageLab software (Bio-Rad). The densitometric signal was background corrected and then normalized to the mean of reference dots for each membrane.

### Human proteases array

To identify the elements of CAKs secretome a Proteases Array Kit (R&D systems) was utilized. The test allows to identify 35 different proteases by using antibodies, which are immobilized on a nitrocellulose membrane. The experiment was performed according to the protocol provided by the manufacturer. Namely, conditioned media collected from: control keratinocytes or treated with CM from A375 or co-cultured with A375 present on Transwell inserts were used. Samples with an equal amount of protein (30 µg) were mixed with a biotinylated detection antibodies cocktail and added onto nitrocellulose membranes containing primary antibody dots and incubated overnight. Further, the membranes were rinsed, and the chemiluminescent signal was detected using streptavidin-HRP. A chemiluminescent signal was measured using ChemiDoc Imaging System (BioRad) and analysed using ImageLab software (Bio-Rad). The densitometric signal was background corrected and then normalized to the mean of reference dots for each membrane.

### qRT-PCR analysis

To measure the expression level of *TIMPs* (tissue inhibitor of metalloproteinases) in CAKs, RNA was isolated using an RNA purification kit (EURx) according to the manufacturer’s instructions. Reverse transcription reaction was conducted using a High-Capacity cDNA Reverse Transcription Kit (Applied Biosystems) following manufacturer’s protocol. Quantitative PCR was performed using PowerUp™ SYBR™ Green Master Mix. Results were normalized to *HPRT1* expression based on the comparative CT (threshold cycle value) method (ΔCT = 2^- (CT gene of interest − CT housekeeping gene). Three biological repetitions each in triplicate were performed. Sequences of primers that were utilized are shown in Table 1.

**Table 1:** Sequences of utilized primers

Gene	Forward primer	Reverse primer
*TIMP1*	gcttctggcatcctgttgttg	acgctggtataaggtggtctg
*TIMP2*	ggtcagtgagaaggaagtggac	gggggccgtgtagataaactc
*TIMP3*	gccttctgcaactccgacatc	cagcttaaggccacagagactc
*HPRT1*	gaccagtcaacaggggacat	gcttgcgaccttgaccatct

### Statistical analysis

All data are given as means ± standard deviation (SD), and their significance was evaluated with GraphPad Prism 7 software applying one-way ANOVA followed by Tukey’s test.

## Results

To investigate the impact of melanoma on HaCaT keratinocytes, we used four melanoma cell lines differing in their origin (primary tumor-derived: WM1341D, A375, and metastatic ones: WM9, Hs294T) and their invasiveness (less invasive WM1341D and highly invasive A375, WM9, and Hs294T), analogous to our recent studies focused on cancer-associated fibroblasts [7].

Due to the fact, that in our previous research we observed varied effects of melanoma cells on fibroblasts depending on the CAFs culturing method [7], in this work we also used two culture systems (Fig. 1). We cultured keratinocytes with melanoma-conditioned media (CM) and co-cultured keratinocytes with melanoma cells present on Transwell inserts (INS). This way we obtained CAKsCM and CAKsINS, respectively.

**Fig. 2:**
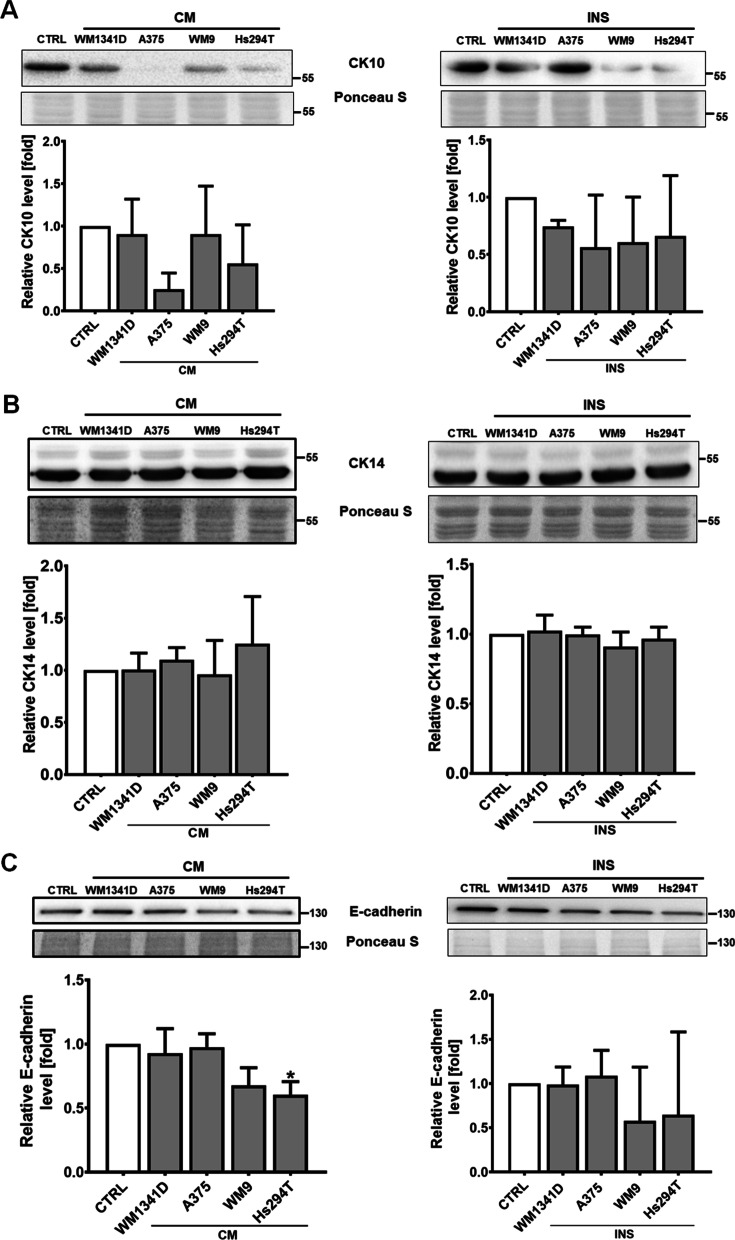
CAKs characterization. The level of CK10 (**A**), CK14 (**B**) and E-cadherin (**C**) in cell lysates was determined using Western Blotting analysis. Cells were cultured in the presence of melanoma on Transwell inserts (INS) or with melanoma-conditioned media (CM). Control (CTRL) constitutes of cells cultured in kDMEM: mDMEM (1:1 ratio) media. Results were normalized to Ponceau S staining. The mean of at least three biological repetitions ± SD is shown. Asterisks indicate statistically significant differences between control cells and CAKs or between different types of CAKs. The significance level was set at p ≤ 0.05 (*)

As mentioned before, under the influence of melanoma cells, keratinocytes express less CK10 and elevated levels of CK14. Considering these findings, we verified CAKs acquisition by estimation of CK10 and CK14 levels in keratinocytes after their incubation with melanoma CM or in co-culture with melanoma present on Transwell inserts. We observed decreased level of CK10, especially in the case of CAKsCM treated with CM collected from A375 and Hs294T, however it was not statistically significant due to high standard deviations (Fig. 2A). The level of CK14 in CAKs was unchanged (Fig. 2B).

As stated before, in physiological conditions keratinocytes control proliferation of melanocytes and prevent their transformation into melanoma, by direct interactions based mainly on E-cadherin. During tumorigenesis, melanoma cells lose expression of E-cadherin in favour of N-cadherin upregulation. In this way, they escape the control of keratinocytes and establish new connections with cells expressing N-cadherin. Due to the important role of E-cadherin in interactions between keratinocytes and melanocytes and thus in its function in cancer development, we evaluated the level of this protein in CAKs. We observed lower E-cadherin levels in CAKs treated with CM collected from WM9 and Hs294T, and CAKs from co-culture with WM9 and Hs294T present on Transwell inserts. However, due to the high standard deviations only the difference between Hs294T CM and control achieved statistic significance (Fig. 2C).

**Fig. 3:**
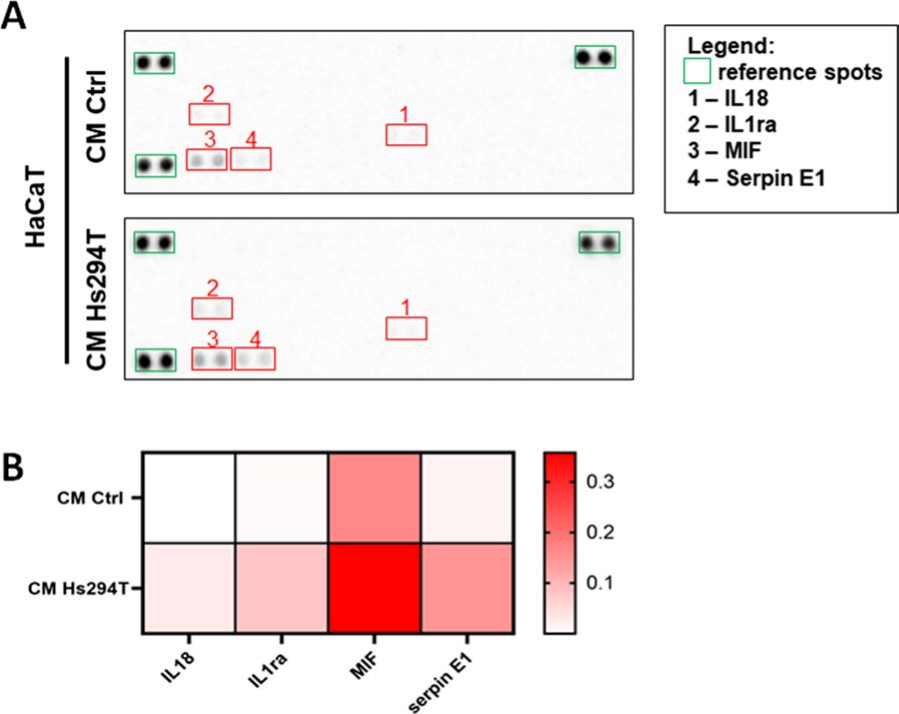
Influence of melanoma on cytokine secretion by keratinocytes. To identify secreted cytokines cell culture media collected from CAKs were used. Based on obtained signal (**A**) quantitative analysis (**B**) was conducted. Results were normalized to reference spots and are shown in a form of heatmap, where darker red indicates a higher intensity of a signal. Abbreviations: IL18, interleukin 18; IL1ra, Interleukin 1 receptor antagonist; MIF, Macrophage migration inhibitory factor

Since previous reports indicate that keratinocytes under the influence of melanoma undergo some functional changes, we decided to further investigate the effect of melanoma on HaCaT keratinocytes, and the potential role played by them in the cancer microenvironment. We started with an analysis of the influence of melanoma on keratinocyte cytokines secretion patterns. We detected a higher level of interleukin 18 (IL18), Interleukin 1 receptor antagonist (IL1ra), Macrophage Migration Inhibitory Factor (MIF), and Serpin E1 in CAKs in comparison to control cells (Fig. 3).

**Fig. 4:**
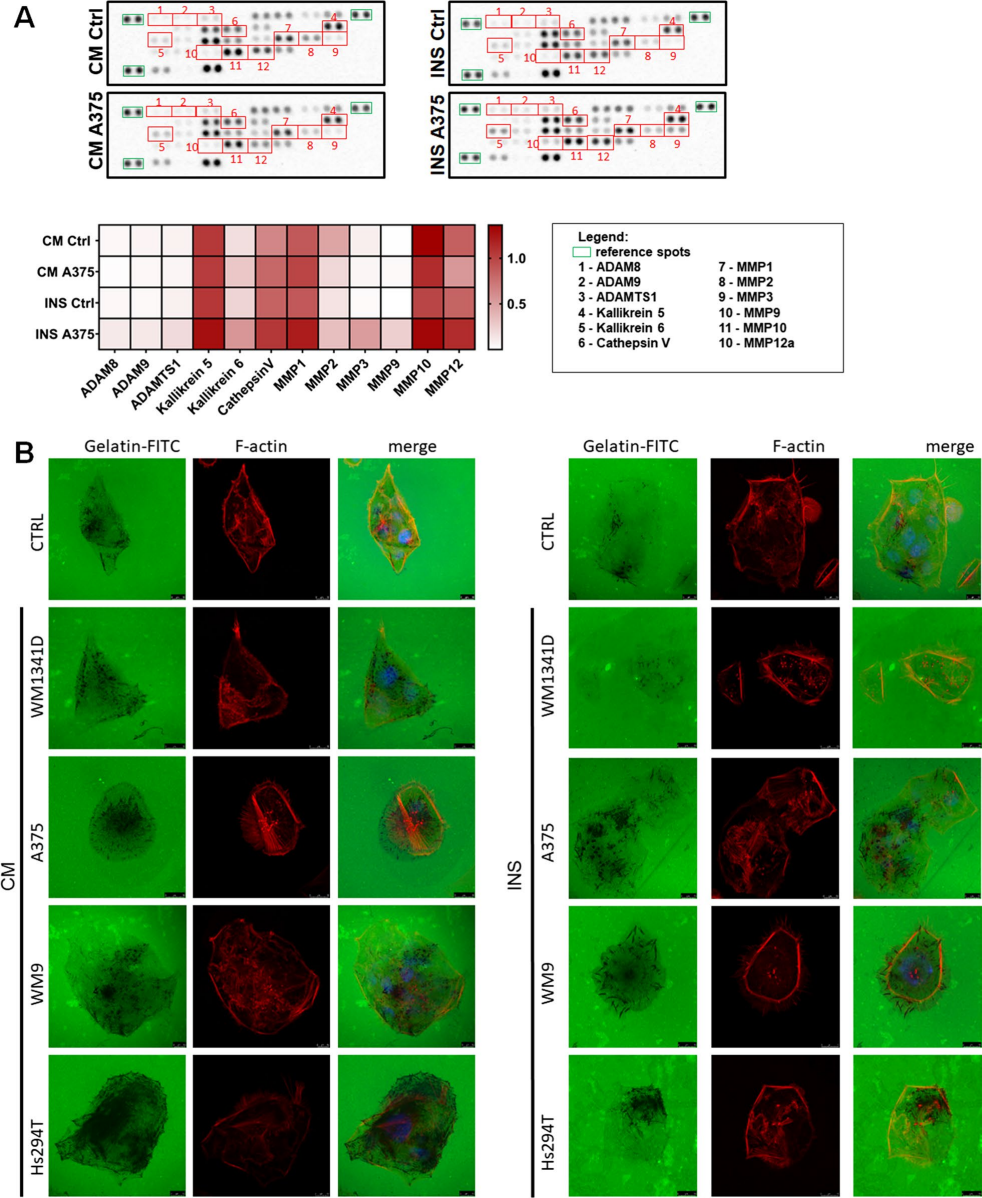
Influence of melanoma on keratinocytes’ proteolytic properties. Secreted by CAKs proteases were identified in conditioned media using proteases array (**A**). Based on obtained signals quantitative analysis was conducted. Results were normalized to reference spots and are shown in a form of heatmap, where darker red indicates a higher intensity of a signal. CAKs’ proteolytic activity was examined also in gelatin-FITC degradation assay (**B**). Cells were cultured in the presence of melanoma on Transwell inserts (INS) or with melanoma-conditioned media (CM). Abbreviations: ADAM, a disintegrin and metalloproteinase; MMPs, matrix metalloproteinases

Moreover, we analysed proteases secreted by CAKs. We found several different proteases, which are secreted by normal keratinocytes and CAKs (Fig. 4A). In the case of CAKsINS, we identified increased levels of: a Disintegrin and metalloproteinase domain-containing protein 8 (ADAM8), ADAM9, a disintegrin and metalloproteinase with thrombospondin motifs 1 (ADAMTs1), kallikrein 5, cathepsin V, matrix metalloproteinases 2 (MMP2), MMP3, MMP9, MMP10 and MMP12, in comparison to control cells. In addition, in both CAKsCM and CAKsINS we detected elevated secretion of kallikrein 6 and MMP1 in comparison to control keratinocytes. The multitude and variety of proteases secreted by CAKs and the effect of melanoma on their level prompted us to analyse the proteolytic activity of these cells. To evaluate the influence of melanoma on proteolytic properties of keratinocytes, we performed a gelatin-FITC degradation assay. We observed higher proteolytic activity in the case of CAKsCM treated with CM collected from Hs294T and CAKsINS co-cultured with highly invasive melanoma cell lines (A375, WM9, Hs294T) in comparison to control cells (Fig. 4B). The data obtained in gelatin-FITC degradation assay are only qualitative, as due to the varied digestion patterns it was impossible to perform quantification.

**Fig. 5:**
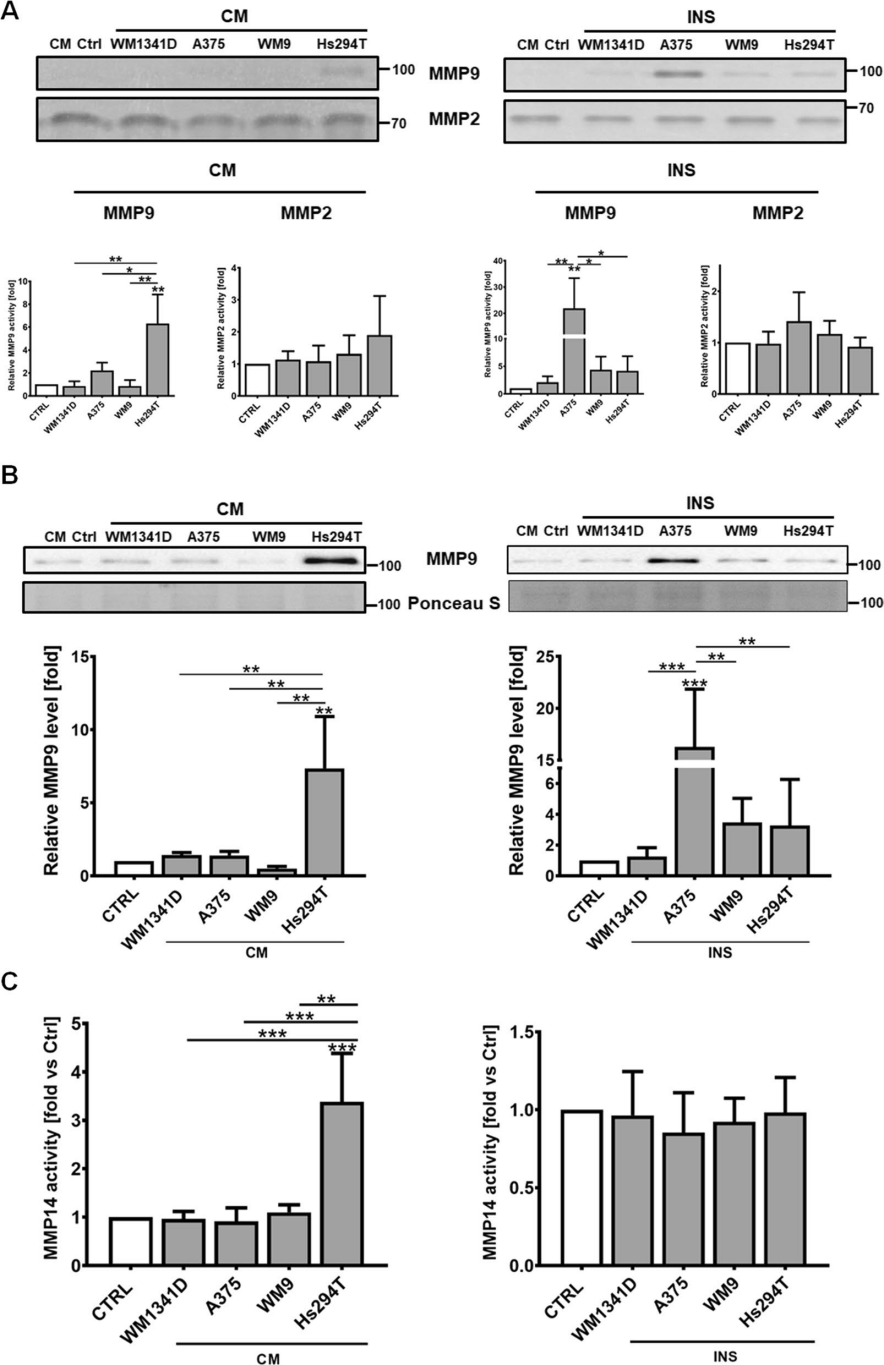
Identification of gelatinases present in CAKs media and MMP14 activity. Zymography analysis of media collected from CAKs (**A**). Western Blotting analysis of MMP9 level in CAKs media (**B**). MMP14 activity assay performed on cell lysates (**C**). Cells were cultured in the presence of melanoma on Transwell inserts (INS) or with melanoma-conditioned media (CM). Control (CTRL) constitutes of cells cultured in kDMEM: mDMEM (1:1 ratio) media analogously to tested cells. In the case of Western Blotting analysis results were normalized to Ponceau S staining. The mean of at least three biological repetitions ± SD is shown. Asterisks indicate statistically significant differences between control cells and CAKs or between different types of CAKs. The significance level was set at p ≤ 0.05 (*), p ≤ 0.01 (**), and p ≤ 0.001 (***)

In order to identify the gelatinases in the CAKs media, which are responsible for the increased degradation of gelatin-FITC, we performed gelatin zymography. We detected a higher level of MMP9 in CAKsCM treated with CM collected from Hs294T and in CAKsINS co-cultured with highly invasive melanoma cell lines (A375, WM9, Hs294T) in comparison to control cells (Fig. 5A). However, in the case of CAKsINS, the statistical significance was achieved only in the case of keratinocytes co-cultured with A375. We also detected the presence of MMP2 in keratinocytes media, however, there was no statistically significant difference between CAKs and control cells. To further confirm the increase in MMP9 level in CAKs media in comparison to control cells, we performed western blot analysis and found elevated MMP9 levels in CAKsCM treated with CM collected from Hs294T, and in CAKsINS co-cultured with A375, WM9 andHs294T (again, with statistical significance only in the case of CAKs cultured with A375) (Fig. 5B). The results of the Western Blotting analysis are consistent with those obtained using the gelatin zymography method.

Subsequently, considering the fact that not all MMPs are secretory enzymes, we decided to check the activity of membrane protease MMP14. We observed higher MMP14 activity in CAKsCM treated with CM collected from Hs294T (Fig. 5C), with no detectable changes in the activity of MMP14 in CAKsINS.

**Fig. 6:**
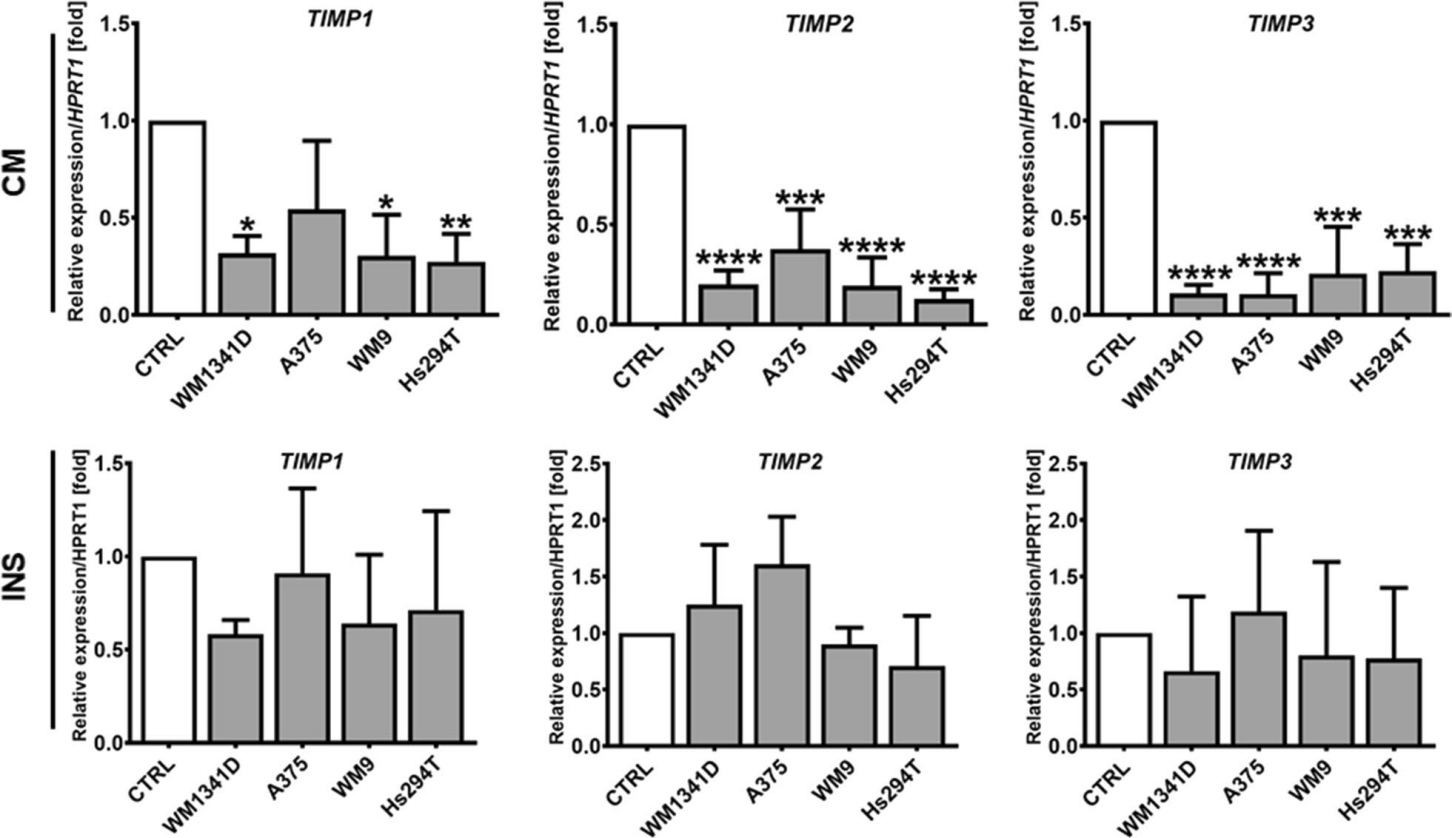
Influence of melanoma on keratinocytes’ expression of TIMPs. Real-time PCR analysis of TIMP1, TIMP2, and TIMP3 expression. Cells were cultured in the presence of melanoma on Transwell inserts (INS) or with melanoma-conditioned media (CM). Control (CTRL) constitutes of cells cultured in kDMEM: mDMEM (1:1 ratio) media analogously to tested cells. The mean of at least three biological repetitions ± SD is shown. Asterisks indicate statistically important differences between control cells and CAKs or between different type of CAKs. The significance level was set at p ≤ 0.05 (*), p ≤ 0.01 (**) and p ≤ 0.001 (***)

Proteolysis might be affected not only by changes in proteases level, but also be altered by the expression of their inhibitors, such as tissue inhibitors of metalloproteinases. Therefore, we analysed the impact of melanoma on TIMPs mRNA expression in CAKs using the real-time PCR method. We have observed decreased levels of TIMP1, TIMP2 and TIMP3 in CAKsCM (Fig. 6). We did not notice significant differences in the case of CAKsINS.

**Fig. 7:**
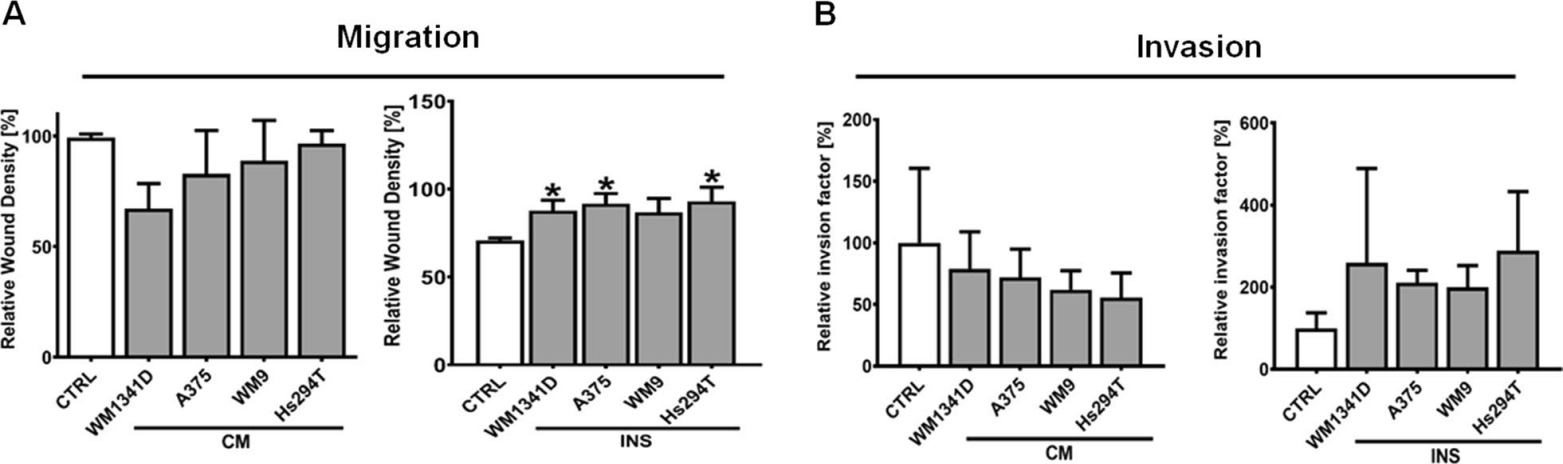
Effect of melanoma cells on migration of keratinocytes (**A**) and invasion (**B**). Cells were cultured in the presence of melanoma on Transwell inserts (INS) or with melanoma-conditioned media (CM). Control (CTRL) constitutes of cells cultured in kDMEM: mDMEM (1:1 ratio) media analogously to tested cells. The mean of at least three biological repetitions ± SD is shown. Asterisks indicate statistically important differences between control cells and CAKs or between different types of CAKs. The significance level was set at p ≤ 0.05 (*)

Proteolytic activity is one of the factors, which contributes to cell motility, as in this way cells form a pathway, which is then used for the invasion to the surrounding tissues. As we detected changes in proteolytic activity appearing in keratinocytes under the influence of melanoma cells, we decided to also analyse the migratory and invasive abilities of CAKs. We identified increased migration of CAKsINS, however, only in the case of CAKs co-cultured with WM1341D, A375, and Hs294T the differences were statistically significant, with no observed changes in the migration of CAKsCM (Fig. 7). In the case of invasion assay, similar to migration, CAKsINS exhibited a tendency for a higher invasion rate (however, it was not statistically significant) and invasion of CAKsCM was not changed. To confirm that increased migration of CAKs is not related to enhanced proliferation of cells, we conducted a proliferation assay and did not detect any significant changes between CAKs and control cells (Additional file 1: Fig. S1).

**Fig. 8:**
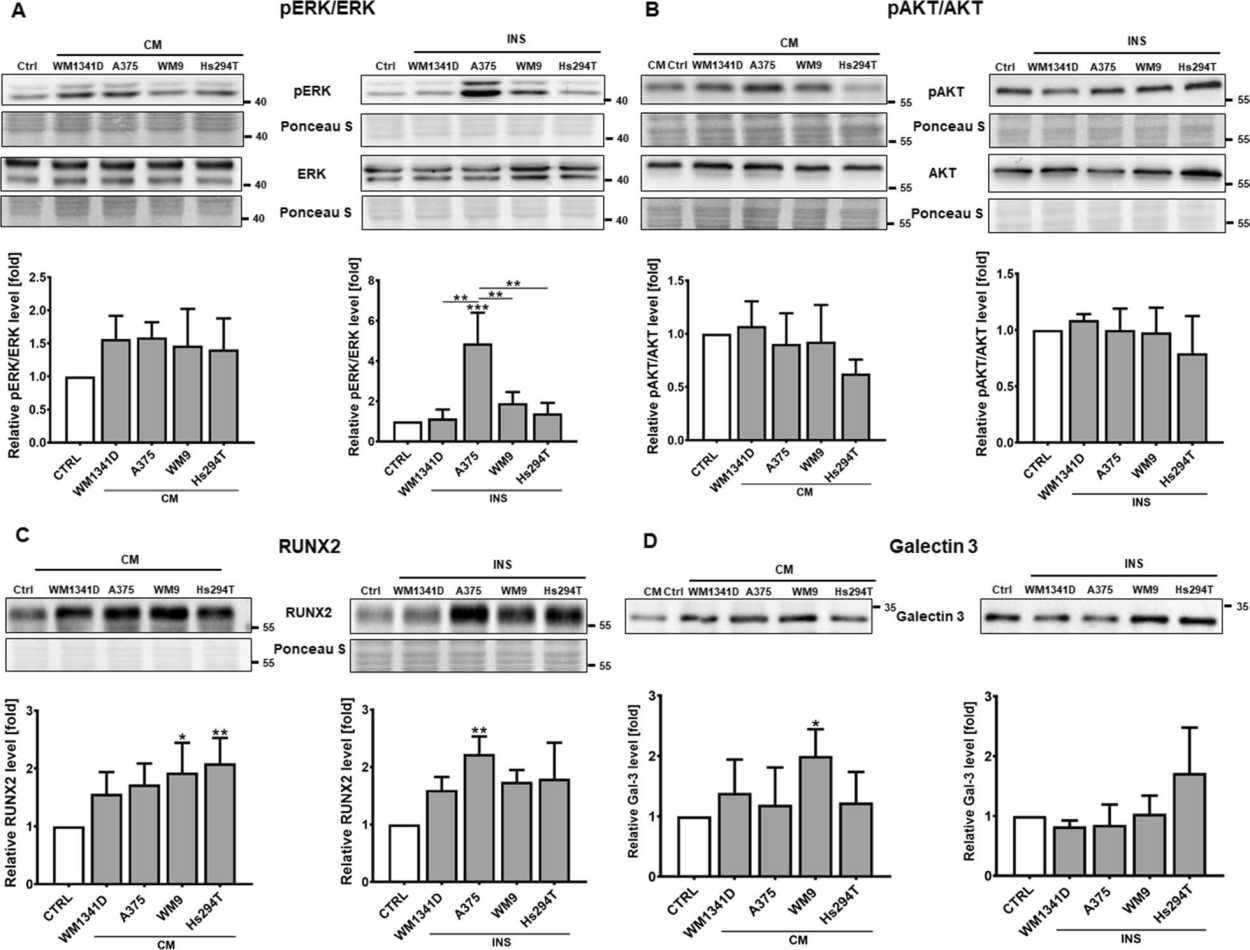
Western Blotting analysis of proteins involved in migration and proteolysis. Cell lysates were used for pERK/ERK (**A**), pAkt/Akt (**B**), and RUNX2 (**C**) levels estimation. Conditioned media collected from HaCaTs were utilized for Galectin 3 level evaluation (**D**). Cells were cultured in the presence of melanoma on Transwell inserts (INS) or with melanoma-conditioned media (CM). Control (CTRL) constitutes of cells cultured in kDMEM: mDMEM (1:1 ratio) media analogously to tested cells. The mean of at least three biological repetitions ± SD is shown. Asterisks indicate statistically important differences between control cells and CAKs or between different type of CAKs. The significance level was set at p ≤ 0.05 (*), p ≤ 0.01 (**) and p ≤ 0.001 (***)

To further unravel the basis of changes in levels of proteases in CAKs, we verified the activation status of MAPK and PI3K/Akt pathways by Western Blotting analyses. We observed increased phosphorylation of ERK kinase in both CAKsCM and CAKsINS, however, it was only statistically significant in the case of CAKsINS, which were co-cultured with A375 (Fig. 8A). We did not observe any significant differences in phosphorylation of Akt kinase in CAKs (Fig. 8B).

Another protein that is linked to MMP expression is runt-related transcription factor 2 (RUNX2) [30]. Since we detected its upregulation in CAFs after melanoma stimulation [7], we decided to also verify its expression level in CAKs. We identified elevated levels of RUNX2 in CAKsCM and CAKsINS, with a statistically significant difference in the case of CAKsCM treated with CM collected from WM9 and Hs294T, and CAKsINS co-cultured with A375 present on Transwell inserts (Fig. 8C). Furthermore, we observed elevated levels of galectin 3 (Gal-3) in CAKs in comparison to control cells, however, only in the case of CAKs cultured in the presence of a conditioned medium collected from WM9 the difference was statistically significant (Fig. 8D). Secreted galectin 3 is known to support cancer cell migration in various ways. Gao et al. have shown, that Gal-3 promotes cell migration by activation of the PKC/ERK pathway [31, 32]. Dange et al. have shown that extracellular Gal-3 induces MMP9 secretion in melanoma cells via the MAPK pathway [32, 33]. Moreover, Gal-3 contributes to tumor angiogenesis. Melanoma cells expressing Gal-3 secrete higher amounts of VEGF than cells with Gal-3 depletion. In in vivo studies, reduced angiogenesis was observed in Gal-3-deficient tumors [34, 35].

## Discussion

The tumor microenvironment is a known factor influencing melanoma progression and affecting its therapy. Among cellular components of melanoma niche keratinocytes are the least studied [36]. There are only a few publications concerning the issue of melanoma’s impact on keratinocytes and changes in keratinocytes, that are arising under the influence of cancer cells [24]. Therefore, to better understand the role of this cellular component in TME, in this study we tried to elucidate, which changes in keratinocytes occur under the impact of melanoma.

In the early stages of our research, we conducted several experiments on the primary keratinocyte cells– HEKa and immortalized one—HaCaT, in a parallel. The primary cells may better reflect in vivo conditions but have a limited number of divisions and are much more difficult to cultivate than immortalized HaCaT. Moreover, primary cells exhibit high heterogeneity between patients and might undergo senescence. Results that we obtained from these two keratinocyte cell types were similar (data comparison not shown). Considering the significant difficulties in the culturing of the primary cells and the consequent lack of success in conducting most of the experiments on HEKa, we decided to limit the experimental conditions to the HaCaT cell line.

As described earlier in the case of research focusing on CAFs [7], here we also used two different methods of CAKs acquisition to get a broader view of the influence of melanoma cells on cancer-associated keratinocytes. We are aware that all in vitro tumor microenvironment models are a significant simplification of the in vivo conditions. We admit that there are models that closer reflect the in vivo conditions, such as 3 D organotypic culture [37] and air–liquid interface [38], however they are based on direct co-culture and do not assume the possibility of subsequent isolation of a given cell type. In our studies, we wanted to evaluate changes in keratinocytes under the influence of melanoma cells, while taking into account modifications in their secretory profile, proteolytic abilities and expression of proteins important for these processes. We would not be able to analyze these features of keratinocytes by handling lysates containing a mixture of melanoma cells and keratinocytes, or media derived from the co-culture of two cell types. Although these methods better reflect the in vivo conditions, they were not suitable for our experiments. Due to all the reasons mentioned above, we decided to use a significantly simplified method of co-culture, while still allowing to study changes in keratinocytes without the need of cell isolation from direct co-culture.

Similar to data obtained on CAFs [7] we observed distinct results from CAKs collected based on two different systems in regard to MMP9 level, MMP14 activity, TIMPs expression, migration, and invasion. One of the methods we utilized was based on the treatment of keratinocytes with melanoma secretome in a form of conditioned media. This model shows greater reproducibility of the results between biological repetitions because a variable factor in this method consists only of keratinocytes, which may undergo changes between different passages during cultivation. The biological replicates of conditioned media are mixed before use, so their content is not overtly variable. The second method constitutes an indirect co-culture system based on the Transwell inserts, where two types of cells are sharing the secretome, however, they are not interacting directly. In the indirect co-culture, the reproducibility of the results may be lower, considering the fact that both kinds of cells, that is the keratinocytes and the melanoma cells, undergo changes of phenotype growing number of passages. However, in this method cells may affect each other through secretome, which better reflects in vivo conditions. Therefore, we found the most appropriate to use both of these methods in our study.

We used four various melanoma cell lines which differ in terms of their origin (two primary tumor-derived: WM1341D, A375, and two metastatic: WM9, Hs294T) and their invasive abilities. The use of a panel of four different melanoma cell lines increases the possibility to make more general observations about their effect on keratinocytes. It is important to note, that keratinocytes located in the skin may interact not only with primary melanoma tumors. Many reports indicate the appearance of recurrences of lesions in about 20% of patients, that form at the site of the scar resulting from excision of the primary tumor [39–42]. Moreover, melanoma metastases within the skin can also occur [43], and the formation of a tumor-promoting niche (including keratinocytes) in the area of the primary tumor can certainly affect the further development of the cancer. We expected that similar to research concerning CAFs [7], in the case of keratinocytes we will identify differences in the influence of primary tumor-derived and metastatic melanoma cell lines on keratinocytes. However, we did not observe such a tendency. We can conclude that this effect is rather cell type-specific than global. Nevertheless, analogous to CAFs [7], HaCaT keratinocytes which were co-cultured with highly invasive melanoma cell lines exhibited higher proteolytic activity (in terms of Gel-FITC degradation analysis and MMP9 level) than control cells as well as and keratinocytes cultured in the presence of less invasive WM1341D.

Our research began with the estimation of the keratins levels as these proteins are considered specific keratinocytes markers that testify to the differentiation status of keratinocytes. We observed decreased CK10 in CAKsCM and CAKsINS, which is consistent with previously published research by Kodet et al. [24]. Moreover, we detected that under the influence of melanoma cells, the level of E-cadherin was reduced in keratinocytes. This protein is crucial for keratinocyte-melanoma interactions, and its restoration in melanoma cells leads to their reattachment to keratinocytes and lower migratory potential of melanoma cells [17]. It is possible that melanoma causes E-cadherin downregulation in keratinocytes to prevent their reconnection, which leads to decrease in cancer cell migration.

Inflammation is an important process in tumor development and progression, thus we decided to unravel, if melanoma affects cytokine secretion pattern of keratinocytes. We found that keratinocytes secrete IL18, IL1ra, MIF and Serpin E1, level of which was increased in keratinocytes incubated with melanoma-conditioned media, as compared to control cells. IL18 is a pro-inflammatory cytokine that promotes the expression of interferon gamma in T cells and has an important role in skin diseases connected to inflammation [44]. Moreover, exogenous IL18 supports melanoma invasion through MAPK activation (with increased ERK phosphorylation) and in a reactive oxygen intermediate (ROI)-depended manner [45]. Keratinocytes are considered a major producer of this interleukin [44]. However, according to our best knowledge, no one has described the enhanced production of IL18 in keratinocytes under the influence of melanoma. MIF is another important pro-inflammatory factor, responsible for maintaining innate immunity, and is also secreted by keratinocytes [46]. Interestingly, the high expression of MIF in clinical studies of melanoma correlates with its more aggressive phenotype [47, 48]. The next protein, which we identified in the CAKs medium was Serpin E1, plasminogen activator inhibitor type-1 (PAI-1). Besides the main function of PAI-1 as a modulator of the plasmin-based proteolytic cascade, it also plays a significant role in cell motility. It interacts with low-density lipoprotein receptor-related protein1 (LRP1) or vitronectin (VN), which attenuates adhesion by altered integrin activation, leading to cell detachment and subsequent migration [49]. Furthermore, a high level of PAI-1 in tumor stroma correlates with a poor prognosis for patients [49, 50]. On the other hand, IL1ra is an inhibitor of IL1. It binds to the IL1 receptor, but does not lead to its activation [51]. Based on our data, it seems that keratinocytes might have a dual role in inflammation, as they secrete both pro- and anti-inflammatory factors.

Due to the low number of cytokines secreted by CAKs, it seems that modulation of inflammatory processes is not their main role. Therefore, we looked further for another possible function of CAKs in the TME. Considering the great role of proteolysis in tumor spreading, we decided to check whether the proteolytic properties of HaCaT keratinocytes change under the influence of melanoma. In a screening analysis, we detected multiple different proteases in CAKs media. Several of them were elevated in CAKsINS e.g., ADAM8, ADAM9, ADAMTS1, Kallikrein 5, Cathepsin V, MMP2, MMP3, MMP9, MMP10 and MMP12, as compared to control cells. The levels of Kallikrein 6 and MMP1 were increased in both CAKsCM and CAKsINS, compared to control keratinocytes.

ADAMs proteases take part in the proteolytic processing of membrane proteins, cell signaling and adhesion. Through proteolysis, they activate proteins, which are produced as precursors such as growth factors, e.g., EGF [52, 53]. Moreover, ADAM8 is involved in neutrophil infiltration, whereas ADAM9, similar to MMP9, is a gelatinase. Interestingly, ADAMTS1 is produced in keratinocytes and fibroblasts during wound healing [52], however according to our knowledge, no one indicated its elevated production in melanoma-associated keratinocytes. Kallikrein 5 is the main serine peptidase present in the skin. Its role in melanoma progression remains unclear, but in gastric adenocarcinoma, it is involved in the migration and invasion of cancer cells [54]. Another protein identified in the CAKs medium was cathepsin V – endopeptidase, which elevated expression correlates with several types of cancer, such as colorectal and breast cancer [55]. In keratinocytes, cathepsin V plays a role in melanosome degradation [56]. Functions of MMPs in ECM remodeling responsible for melanoma invasion have been extensively studied [57]. MMP3 is known to be expressed by keratinocytes [58] and to support melanoma metastasis formation [59], however its elevated expression in CAKs was not described before. Moreover, MMP9 was shown to be secreted by keratinocytes under the influence of melanoma in the reconstructed skin model, which is consistent with our findings [60]. Furthermore, we demonstrated higher kallikrein 6 levels in CAKs, which is in line with the findings of Krenzer et al., who described that it is produced by melanoma-adjacent keratinocytes, and supports tumor cell migration and invasion [61].

As we detected several different proteases in CAKs media, and some of them were changed under the influence of melanoma cells, we decided to investigate, if CAKs proteolytic activity is also altered. We showed that CAKs degrade gelatin-FITC to a higher extent in comparison to control cells which is probably caused by an elevated level of MMP9 and MMP14 in CAKs. Enhanced proteolysis in CAKs is also supported by decreased expression of TIMP1, TIMP2, and TIMP3. Based on our knowledge of previously published literature, these findings are novel. Higher proteolytic activity of keratinocytes growing in tumor niche might lead to enhanced migration of cancer cells, as in this way cells create a pathway, which is then used to move through the tissue [62].

Moreover, CAKs exhibited increased motility abilities, which is one of cell features involved in many processes such as wound healing, indicated to be important for ulcers healing, that appear in melanoma patients [63]. We hypothesize, to the possibility, that increased migration of CAKs have impact on ulceration healing in melanoma. Moreover, enhanced motility of keratinocytes combined with their increased proteolysis might facilitate invasion of melanoma cells.

Next, to elucidate the basis of changes in proteolytic activity and migration of CAKs, we analysed MAPK pathway activation and detected enhanced phosphorylation of ERK, which might lead to elevated migration and proliferation of these cells and, in this way, support melanoma progression [64]. We also detected a higher level of transcription factor-RUNX2 involved in expression of MMPs, upregulation of which we previously observed in CAFs [7, 65]. A higher level of Galectin 3 in CAKs, in comparison to control keratinocytes, which we have also demonstrated, has not been previously shown and might contribute to increased cancer cell movement through activation of the PKC/ERK pathway [66].

## Conclusions

In conclusions, the utilization of two various models that mimic the influence of melanoma cells on cancer-associated keratinocytes within TME is necessary for a broader view of the relationship between these cell types. Melanoma leads to the increased proteolytic activity of CAKs, which might be caused by enhanced MAPK pathway activation or RUNX2 elevated level in CAKs. Moreover, we observed that under the influence of melanoma, HaCaT keratinocytes secrete higher amounts of galectin 3, which is involved in cell migration. Increased proteolytic activity of CAKs and secretion of galectin 3 might impact cancer progression. The study of relation between melanoma cells and keratinocytes is important, as it extends the knowledge about the role of TME in melanoma spreading.

## Supplementary Information

**Additional file 1. Fig. S1** Influence of melanoma cells on CAKs proliferation. Cells were cultured in the presence of melanoma on Transwell inserts (INS) or with melanoma-conditioned media (CM). Control (CTRL) constitutes of cells cultured in kDMEM: mDMEM (1:1 ratio) media analogously to tested cells. The mean of at least three biological repetitions ± SD is shown.

## Data Availability

The datasets during and/or analyzed during the current study are available from the corresponding author on reasonable request.

## Abbreviations

ADAM8: A Disintegrin and metalloproteinase domain-containing protein 8
ADAMTs1: A disintegrin and metalloproteinase with thrombospondin motifs 1
CAKs: Cancer-associated keratinocytes
CK10: Cytokeratin 10
CM: Conditioned media
CT: Threshold cycle value
DMEM: Dulbecco’s Modified Eagle Medium
EDTA: Ethylenediaminetetraacetic acid
End-1: Endothelin-1
ERK: Extracellular-signal regulated kinase
FBS: Fetal bovine serum
Gal-3: Galectin 3
HPRT1: Hypoxanthine–guanine-xanthine phosphoribosyltransferase
IL18: Interleukin 18
IL1ra: Interleukin 1 receptor antagonist
INS: Transwell inserts
KGF: Keratinocyte growth factor
LRP1: Low-density lipoprotein receptor-related protein1
MAPK: Mitogen-activated protein kinase
MIF: Macrophage Migration Inhibitory Factor
MITF: Melanocyte inducing transcription factor
MMP: Matrix metalloproteinase
PAI-1: Plasminogen activator inhibitor type-1
PBS: Phosphate-buffered saline
PKC: Protein kinase C
RUNX2: Runt-related transcription factor 2
SCF: Stem cell factor
SD: Standard deviation
TIMP: Tissue inhibitor of metalloproteinases
TME: Tumor microenvironment
VN: Vitronectin
