# Supplementary figures and images for: Melanoma stimulates the proteolytic activity of HaCaT keratinocytes
Source: Cell Commun Signal. 2022 Sep 19;20:146. doi: 10.1186/s12964-022-00961-w (PMC9484146; doi:10.1186/s12964-022-00961-w)

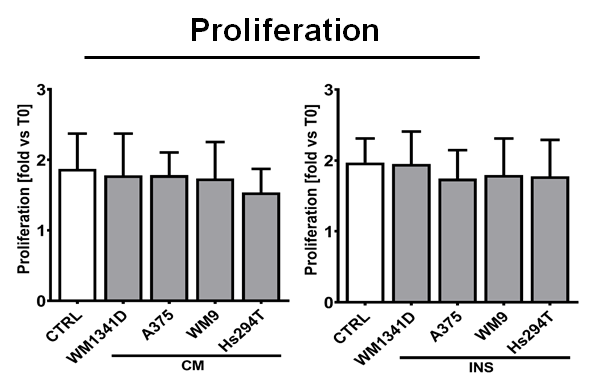

Supplement: Supplementary file 2 — Additional file 1. Fig. S1 Influence of melanoma cells on CAKs proliferation. Cells were cultured in the presence of melanoma on Transwell inserts (INS) or with melanoma-conditioned media (CM). Control (CTRL) constitutes of cells cultured in kDMEM: mDMEM (1:1 ratio) media analogously to tested cells. The mean of at least three biological repetitions ± SD is shown. [file 12964_2022_961_MOESM2_ESM.tif]
